# Nighttime intensivist staffing and the timing of death among ICU decedents: a retrospective cohort study

**DOI:** 10.1186/cc13033

**Published:** 2013-10-03

**Authors:** Lora A Reineck, David J Wallace, Amber E Barnato, Jeremy M Kahn

**Affiliations:** 1 Division of Pulmonary, Allergy and Critical Care Medicine, University of Pittsburgh School of Medicine, 3459 Fifth Avenue, 628 NW, Pittsburgh, PA 15213, USA; 2 Department of Critical Care Medicine, Clinical Research, Investigation and Systems Modeling of Acute Illness (CRISMA) Center, University of Pittsburgh School of Medicine, 3550 Terrace Street, Pittsburgh, PA 15261, USA; 3 Department of Emergency Medicine, University of Pittsburgh School of Medicine, Suite 10028 Forbes Tower, Pittsburgh, PA 15260, USA; 4 Division of General Internal Medicine, University of Pittsburgh School of Medicine, UPMC Montefiore Hospital, Suite W933 200 Lothrop Street, Pittsburgh, PA 15213, USA; 5 Department of Health Policy and Management, University of Pittsburgh Graduate School of Public Health, 130 De Soto Street, Pittsburgh, PA 15261, USA

## Abstract

**Introduction:**

Intensive care units (ICUs) are increasingly adopting 24-hour intensivist physician staffing. Although nighttime intensivist staffing does not consistently reduce mortality, it may affect other outcomes such as the quality of end-of-life care.

**Methods:**

We conducted a retrospective cohort study of ICU decedents using the 2009–2010 Acute Physiology and Chronic Health Evaluation clinical information system linked to a survey of ICU staffing practices. We restricted the analysis to ICUs with high-intensity daytime staffing, in which the addition of nighttime staffing does not influence mortality. We used multivariable regression to assess the relationship between nighttime intensivist staffing and two separate outcomes potentially related to the quality of end-of-life care: time from ICU admission to death and death at night.

**Results:**

Of 30,456 patients admitted to 27 high-intensity daytime staffed ICUs, 3,553 died in the hospital within 30 days. After adjustment for potential confounders, admission to an ICU with nighttime intensivist staffing was associated with a shorter duration between ICU admission and death (adjusted difference: –2.5 days, 95% CI -3.5 to -1.5, p-value < 0.001) and a decreased odds of nighttime death (adjusted odds ratio: 0.75, 95% CI 0.60 to 0.94, p-value 0.011) compared to admission to an ICU without nighttime intensivist staffing.

**Conclusions:**

Among ICU decedents, nighttime intensivist staffing is associated with reduced time between ICU admission and death and reduced odds of nighttime death.

## Introduction

Intensivist physician staffing is associated with lower mortality and decreased length of stay in the ICU [1,2]. This observation has led to efforts to expand the intensivist physician staffing model [3], not only during the day but also at night [4,5]. However, the data in support of nighttime intensivist staffing are not as robust as those for daytime intensivist staffing. Two observational before-and-after studies showed mixed results [6,7], and both a recent multicenter observational study and a single-center randomized trial concluded that nighttime intensivist staffing does not influence mortality in ICUs with high-intensity daytime staffing (defined as a mandatory intensivist consult or closed ICU) [8,9].

Although nighttime intensivist staffing may not reduce mortality in all ICUs, extending intensivist coverage around the clock may have other benefits, including an effect on the quality of end-of-life care. The quality of end-of-life care is an important patient-centered outcome and may be impacted by nighttime intensivist staffing through earlier and more frequent conversations between physicians and surrogate decision-makers regarding prognosis and goals of care [10]. Since intensive communication leads to earlier decisions regarding life-sustaining therapy withdrawal [11,12], increased communication through the addition of nighttime intensivists could result in earlier withdrawal of life-sustaining therapy and thereby alter the timing of death among ICU decedents.

The purpose of this study was to determine whether nighttime intensivist staffing is associated with the timing of deaths in ICU decedents. We examined two complementary elements of the timing of death: the duration between ICU admission and death, and the odds of death at night. The duration between ICU admission and death is often considered a proxy for the quality of end-of-life care, since reduced hospital and ICU stays for decedents without an increase in overall mortality represents a decrease in ineffective aggressive therapy and prolonged dying [10]. The odds of death at night may serve as a proxy for the timing of life-sustaining therapy withdrawal since the median time from terminal withdrawal of mechanical ventilation to death in the ICU is less than 1 hour [13], indicating more timely action in response to decisions to withhold life-sustaining therapy. We hypothesized that, among ICU decedents, nighttime intensivist staffing would be associated with a reduced length of time between ICU admission and death and increased odds of death at night.

## Materials and methods

### Study design

We performed a retrospective cohort study of ICU decedents using the Acute Physiology and Chronic Health Evaluation (APACHE) clinical information system (Cerner, Kansas City, MO, USA) from 2009 through 2010. APACHE collects detailed clinical, physiological, and outcome data on adult ICU patients at participating hospitals for benchmarking and quality improvement. The APACHE database has been used for numerous observational studies involving critically ill patients [14,15]. We linked these data to a 2010 survey conducted in APACHE ICUs that included questions regarding ICU organization, providers, and protocols, as previously described [8].

### Patients

Patients who were 17 years of age or older and admitted to a study ICU were eligible for inclusion in the study. We excluded readmissions, patients admitted to low-intensity ICUs, those who survived to hospital discharge, and decedents whose death occurred more than 30 days after ICU admission. We limited the study to ICUs with high-intensity daytime staffing, for which nighttime intensivist staffing does not affect mortality [8], to eliminate the competing risk of death as a determinant of length of stay. We excluded patients whose death occurred more than 30 days after ICU admission to ensure that our results were not driven by long-stay outliers.

### Variables

The two primary outcome variables were time from ICU admission to death and odds of death at night (defined as death occurring between 7:00 pm and 7:00 am). These variables were chosen because they may represent proxies for the quality of end-of-life care [10] and the timing of life-sustaining therapy withdrawal [13]. The primary exposure variable was presence or absence of a nighttime intensivist, defined as an intensivist attending physician who was physically present in the ICU or elsewhere in the hospital and was immediately available to manage ICU emergencies during nighttime hours [8].

Covariates were specified *a priori* as potential confounders between nighttime staffing and timing of death based on previous studies [14,15]. Patient-level covariates included age, race, sex, Acute Physiology Score (a measure of the severity of illness ranging from 0 to 252, with higher scores indicating more severe illness and a higher risk of death), the presence or absence of selected coexisting conditions (the acquired immunodeficiency syndrome, leukemia or myeloma, lymphoma, cirrhosis, liver failure, immunosuppression, and metastatic cancer), the location of the patient before admission to the ICU (emergency department, operating room, hospital floor, other hospital, or other location), the length of the hospital stay before ICU admission, the admission diagnosis, the patient’s need for emergency surgery, and the patient’s receipt of invasive mechanical ventilation at the time of admission [15]. We also included the teaching status of the hospital determined by the ratio of residents to beds (with a ratio of 0 indicating a nonteaching hospital, 0 to <0.25 a minor teaching hospital, and ≥0.25 a major teaching hospital), the annualized ICU volume of admissions, and type of ICU (specialty or mixed) [16].

### Statistical analysis

We compared the characteristics of hospitals with and without nighttime intensivist staffing using the Mann–Whitney test for continuous variables and Fisher’s exact test for categorical variables. We compared patient characteristics between ICUs with and without nighttime intensivist staffing using two-sample *t* tests for continuous variables and chi-square tests for categorical variables.

Unadjusted time from ICU admission to death was evaluated using Kaplan–Meier curves. Differences in the survivor functions between decedents admitted to ICUs with and without nighttime intensivist staffing were evaluated using the log-rank test.

We used multivariable linear regression to assess the relationship between nighttime intensivist staffing and time from ICU admission to death. We chose linear regression of untransformed length of stay over other approaches since it is a valid method for large samples regardless of distribution, including highly non-normal samples of >500 subjects, when the mean value is the outcome of interest [17]. We used multivariable logistic regression to assess the relationship between nighttime intensivist staffing and the odds of death at night. In all models, generalized estimating equations were used to account for clustering at the level of the ICU [18].

Patient race was missing in 9% of our cohort, and this variable is known to relate to the intensity of end-of-life care for hospitalized patients [19]. To account for missing race we performed multiple imputations using multinomial logistic regression [20,21], creating 10 imputed datasets. Multiple imputation was performed on the entire cohort of patients (including decedents and patients who survived to hospital discharge admitted to either low-intensity or high-intensity daytime staffed ICUs), excluding readmissions. Patient variables used for imputation included the patient-level covariates mentioned above as well as the patient’s discharge disposition (dead, hospice, or alive), length of time from ICU admission to death, whether or not the patient underwent coronary artery bypass graft surgery, whether or not the patient underwent an operation, whether or not the patient received active therapy on ICU admission [22], the presence or absence of diabetes, and whether or not the patient was admitted to the ICU at nighttime (7:00 pm to 7:00 am). All analyses were performed separately on each of the 10 imputed datasets, and the results were combined using Rubin’s rules [23].

### Subgroup analyses

We repeated our analyses in *a priori* selected subgroups with which we hypothesized that nighttime intensivists would interact most frequently, thereby having the largest effect on the timing of death. These subgroups included patients admitted to the ICU at night and two subgroups of patients likely to decompensate overnight due to their increased severity of illness: patients mechanically ventilated on ICU admission and patients with an Acute Physiology Score ≥75 (corresponding to an approximate 25% predicted risk of in-hospital death [15]).

### Sensitivity analysis

We performed a number of sensitivity analyses to assess the robustness of our findings. First, we repeated our analyses using ICU length of stay rather than time from ICU admission to death, since the time from ICU admission to death may be affected by the timing of transfer out of the ICU for patients ultimately dying on the ward. Second, we repeated the time to death analysis considering patients discharged to a hospice as decedents, since the decision to die in the hospital versus a hospice may be affected by patient and surrogate preferences. Third, we examined the effect of nighttime intensivists on the odds of death at night using a second definition of death at night (8:00 pm to 8:00 am) in order to account for the time delay between withdrawal of life-sustaining therapy and death [13].

All statistical analyses were performed using Stata 12.0 (StataCorp, College Station, TX, USA). All tests were two tailed and *P* ≤0.05 was considered significant. This research was approved by the Institutional Review Board of the University of Pittsburgh. A waiver for individual informed consent was provided due to the minimal risk for participants.

## Results

**Figure 1:**
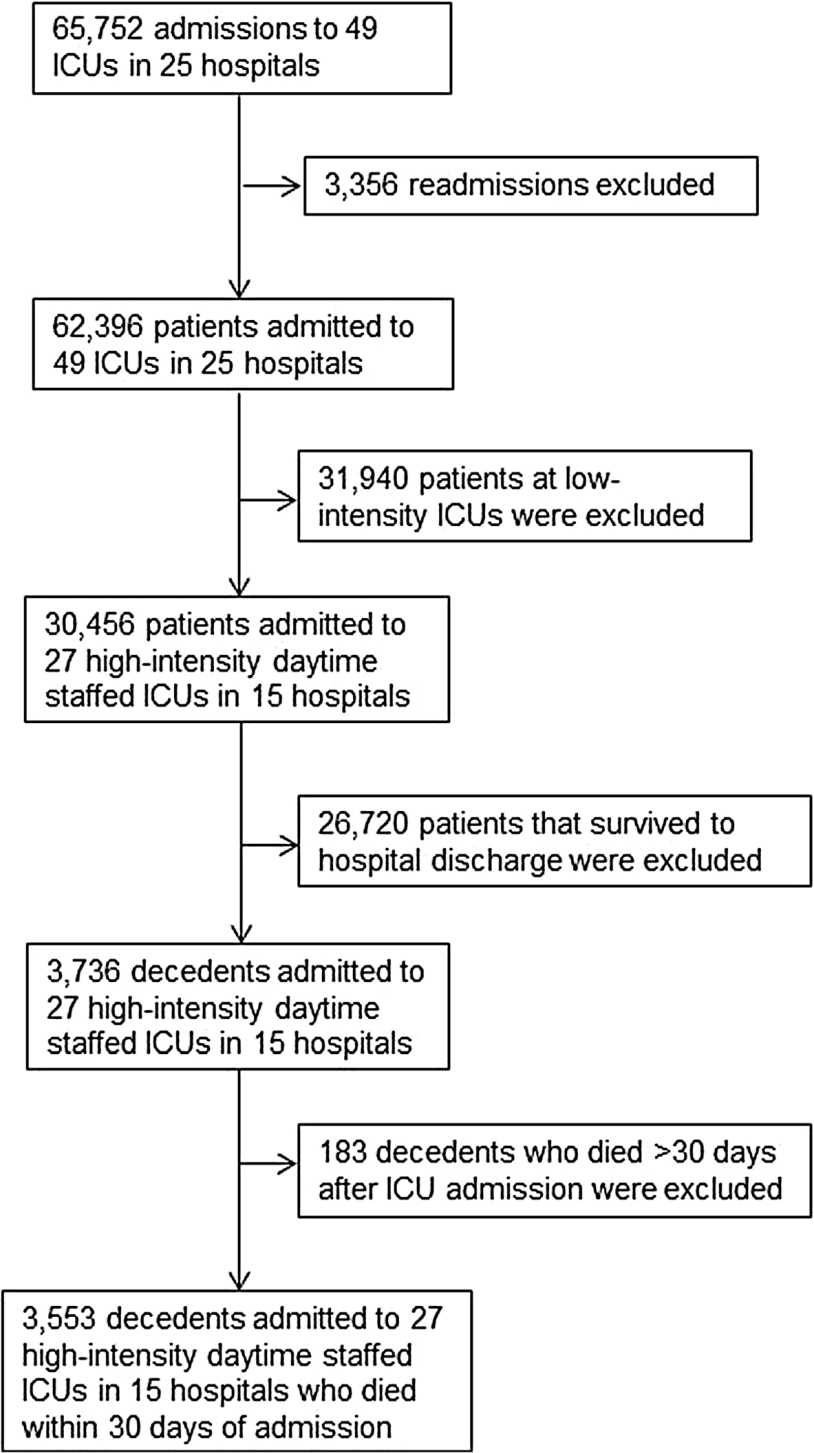
Number of decedents, ICUs, and hospitals in the study.

The initial cohort included 64,752 admissions to 49 ICUs in 25 hospitals. A total of 3,553 decedents admitted to 27 high-intensity daytime staffed ICUs in 15 hospitals met the study inclusion criteria (Figure 1). As previously reported, the odds ratio for in-hospital death in this cohort’s high-intensity daytime staffed ICUs with nighttime staffing compared with the ICUs without nighttime intensivist staffing was 1.08 (95% confidence interval, 0.63 to 1.84, *P* = 0.78) [8]. Six ICUs with nighttime intensivists contributed data on 863 decedents, and 21 ICUs without nighttime intensivists contributed data on 2,690 decedents. Characteristics of the 27 ICUs in the study are shown in Table 1. The ICUs without nighttime intensivists more commonly had a full-time ICU director and were located within a large hospital. Other characteristics of the ICUs were similar.

**Table 1:** Characteristics of high-intensity daytime staffed ICUs

**Characteristic**	**Nighttime intensivists (*n *** **= 6)**	**No nighttime intensivists (*n *** **= 21)**	***P *** **value**
Annualized ICU admissions	517 (418 to 603)	770 (514 to 979)	0.24
ICU type			>0.99
Mixed	3 (50)	11 (52)	
Specialty	3 (50)	10 (48)	
Full-time physician ICU director	2 (33)	20 (95)	0.004
Routine participation of medical students, residents, or other physician trainees	5 (83)	21 (100)	0.22
Daily multidisciplinary rounds	6 (100)	17 (81)	0.55
Hospital beds			0.02
<250	1 (17)	0 (0)	
250 to 500	4 (67)	5 (24)	
>500	1 (17)	16 (76)	
Academic status^a^			0.42
Major teaching	5 (83)	12 (57)	
Minor teaching	0 (0)	6 (29)	
Nonteaching	1 (17)	3 (14)	
Region			0.63
Midwest	4 (67)	9 (43)	
Northeast	0 (0)	3 (14)	
Southeast	1 (17)	7 (33)	
West	1 (17)	2 (10)	

Data presented as median (interquartile range) or *n* (%).

^a^Teaching status categorized by resident-to-bed ratio (nonteaching, 0; minor teaching, >0 to <0.25; major teaching, ≥0.25).

**Figure 2:**
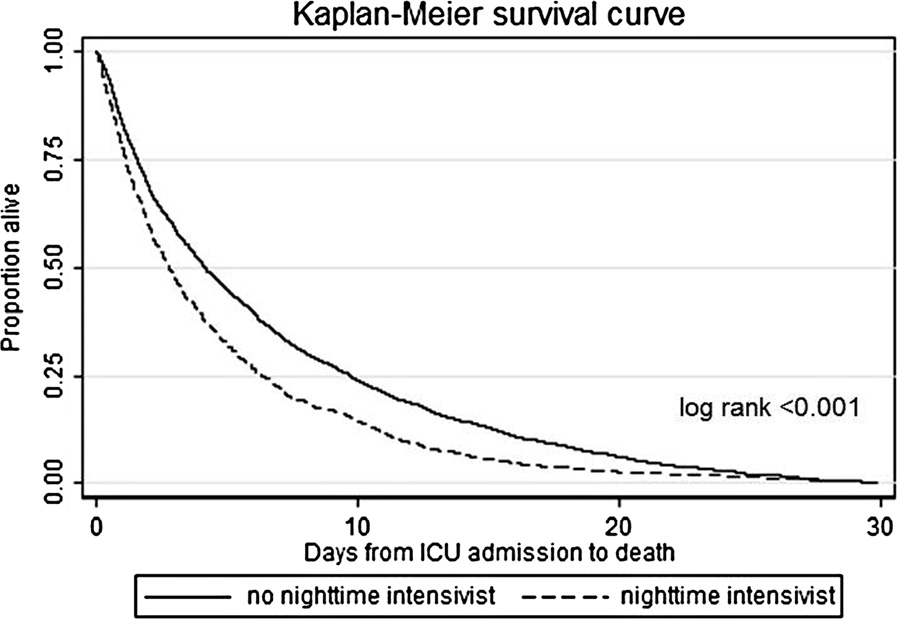
**Kaplan–Meier curve for time from ICU admission to death.** Time from ICU admission to death (days) for decedents admitted to ICUs with nighttime intensivists compared with ICUs without nighttime intensivists.

Characteristics of the 3,553 decedents in the study are shown in Table 2. Decedents admitted to ICUs with nighttime intensivists had a shorter time between ICU admission and death, a shorter ICU length of stay, and fewer nighttime deaths compared with those admitted to ICUs without nighttime intensivists. A Kaplan–Meier survival curve demonstrated that the unadjusted time from ICU admission to death was significantly shorter in ICUs with nighttime intensivists compared with those without through 30 days of follow-up (Figure 2, log-rank *P* <0.001).

**Table 2:** **Characteristics of decedents in high-intensity daytime staffed ICUs**^**a**^

**Characteristic**	**ICUs with nighttime intensivists (*n *** **= 863)**	**ICUs without nighttime intensivists (*n *** **= 2,690)**	***P *** **value**
Age (years)	71 (58 to 80)	68 (56 to 79)	0.001
Female sex	393 (45.5)	1229 (45.7)	0.94
Race			<0.001
White	719 (83.3)	2009 (74.7)	
Black	29 (3.4)	301 (11.2)	
Other	113 (13.1)	70 (2.6)	
Data missing	2 (0.2)	310 (11.5)	
Admission source			<0.001
Emergency department	331 (38.4)	1145 (42.6)	
Operating room	77 (8.9)	418 (15.5)	
Medical or surgical ward	221 (25.6)	769 (28.6)	
Transfer	223 (25.8)	286 (10.6)	
Other	11 (1.3)	72 (2.7)	
Reason for ICU admission			<0.001
Surgery	64 (7.4)	333 (12.4)	
Cardiac disorder	102 (11.8)	233 (8.7)	
Respiratory disorder	199 (23.1)	411 (15.3)	
General medical disorder	49 (5.7)	145 (5.4)	
Sepsis	92 (10.7)	511 (19.0)	
Trauma	23 (2.7)	187 (7.0)	
Neurosurgery	11 (1.3)	44 (1.6)	
Cardiac arrest	122 (14.1)	291 (10.8)	
Other	201 (23.3)	535 (19.9)	
Acute physiology score	63 (45 to 85)	71 (51 to 97)	<0.001
Active treatment on day of ICU admission^b^	749 (86.8)	2397 (89.1)	0.06
Mechanical ventilation on day of ICU admission	643 (74.5)	2028 (75.4)	0.60
Emergency surgery	29 (3.4)	183 (6.8)	<0.001
Length of hospital stay before ICU admission (days)	0 (0.0 to 0.0)	0.2 (0.0 to 1.5)	<0.001
ICU length of stay (days)	2.7 (1.1 to 6.0)	3.0 (1.3 to 6.8)	<0.001
Length of time from ICU admission to death (days)	2.8 (1.1 to 6.3	4.1 (1.5 to 9.7)	<0.001
Deaths at night^c^	309 (35.8)	1130 (42.0)	0.001

Data presented as median (interquartile range) or *n* (%).

^a^Patient characteristics in this table are those that were observed, not imputed or adjusted.

^b^Active treatment was defined as any of 33 active life-supporting intensive care treatments [21].

^c^Night defined as 7:00 pm to 7:00 am.

In the multivariable analysis, we found that adjusted length of time from ICU admission to death was significantly shorter among decedents admitted to ICUs with nighttime intensivists compared with those without (Table 3). This was also true for each of the subgroups analyzed. The adjusted odds of death at night was significantly lower among decedents admitted to ICUs with nighttime intensivists compared with those admitted to ICUs without nighttime intensivists (Table 4). The difference in the odds of death at night was not significant in any of the subgroups, although the odds ratios were similar to those of the full cohort. The sensitivity analyses yielded similar results (Additional file 1: Table S1).

**Table 3:** Adjusted time from ICU admission to death for ICUs with versus without nighttime intensivists

**Cohort**	***N***	**Days (95% CI)**	***P *** **value**
Complete cohort	3,553	-2.5 (-3.5 to –1.5)	<0.001
Subgroups			
Patients admitted to the ICU at night	1,647	-3.0 (-4.0 to –1.9)	<0.001
Patients mechanically ventilated on ICU admission	2,671	-2.1 (-2.9 to –1.2)	<0.001
Patients with Acute Physiology Score ≥75	1,551	-1.6 (-2.5 to –0.7)	0.001

CI, confidence interval.

**Table 4:** Adjusted odds ratio for death at night in ICUs with versus without nighttime intensivists

**Cohort**	***N***	**Odds ratio (95% CI)**	***P *** **value**
Complete cohort	3,553	0.75 (0.60 to 0.94)	0.01
Subgroups			
Patients admitted to the ICU at night	1,647	0.76 (0.56 to 1.03)	0.08
Patients mechanically ventilated on ICU admission	2,671	0.81 (0.62 to 1.05)	0.11
Patients with Acute Physiology Score ≥75	1,551	0.95 (0.68 to 1.33)	0.76

CI, confidence interval.

## Discussion

In a retrospective cohort study of decedents admitted to high-intensity daytime staffed ICUs, the presence of a nighttime intensivist was associated with a decrease in time from ICU admission to death by an average of 2.5 days. This finding suggests that the addition of nighttime intensivists to high-intensity daytime staffed ICUs may improve the quality of end-of-life care for ICU decedents. A possible mechanism may be earlier and more frequent conversations between intensivists and surrogates regarding prognoses and goals of care, leading to earlier withdrawal of life-sustaining treatment. Nighttime intensivist staffing may improve both the timing of communication with surrogates and surrogates’ access to physicians, which research suggests are two aspects of end-of-life care in need of improvement [24]. As a result, decisions to limit life-sustaining therapy may occur earlier in the ICU course [11,12], resulting in earlier transition to palliative care for patients and increased family and clinician satisfaction [25].

Contrary to our hypothesis, we found that the addition of a nighttime intensivist to high-intensity daytime staffed ICUs was associated with decreased odds of death at night. Although nighttime intensivist staffing may lead to earlier withdrawal of life-sustaining treatment, this finding suggests that it is unlikely to be occurring more frequently at night. Instead, nighttime intensivists may have discussions with surrogates preparing them for withdrawal of life-sustaining treatment by the daytime team. Additionally, the decreased odds of death at night may indicate that nighttime intensivists are better able to manage decompensating patients overnight than non-intensivists, forestalling death in the short term. Fewer deaths at night may allow more family members to be present at the time of life-sustaining treatment withdrawal and patient death, further supporting the notion that nighttime intensivists may improve the quality of end-of-life care for ICU decedents [26].

Our findings have implications for the many ICUs that have already implemented nighttime intensivist staffing or those that are considering implementing nighttime staffing. A relationship between nighttime intensivists and the quality of end-of-life care for ICU decedents may provide a rationale for the continuation or implementation of the nighttime intensivist ICU staffing model, even in the absence of a mortality benefit. Additionally, the reduction in time between ICU admission and death represents decreased resource utilization.

Our study has several limitations. The hospitals in this study were a self-selected group of hospitals participating in the APACHE clinical information system rather than a random sample. Compared with other hospitals in the United States, APACHE hospitals are generally larger and more likely to be affiliated with an academic institution. Therefore it is not known whether the findings in this study would be applicable to all other high-intensity daytime staffed ICUs. Second, we acknowledge that confounding by unmeasured ICU characteristics may have affected our results. For example, ICUs with nighttime intensivist staffing may have different norms regarding the use of time-limited trials of life-sustaining treatment for patients than ICUs without nighttime intensivist staffing; this could be an alternate explanation for the reduced time between ICU admission and death. Third, we did not directly measure the quality of end-of-life care; we used a proxy. We acknowledge that there is limited evidence regarding the relationship between timing of death and the quality of end-of-life care, and that a shorter duration between ICU admission and death may not be associated with higher family satisfaction in all cases [27]. Future work should directly examine the relationship between nighttime staffing and the quality of end-of-life care. Finally, we did not assess the behavior of nighttime intensivists in terms of their communication with patients and families, the timing of patient or family decisions regarding life-sustaining treatment, or whether or not death occurred following a decision to withdraw life-sustaining treatment. These variables were not available in our data. Future work should take a more granular approach and examine how nighttime intensivists and surrogates interact and when treatment decisions occur. This would allow a better understanding of the true mechanisms of our findings and help uncover ways to optimize the role of nighttime intensivists in the dying process.

## Conclusions

Among ICU decedents, the addition of nighttime intensivist staffing to high-intensity daytime staffing is associated with a reduced time between ICU admission and death and reduced odds of death at night.

## Key messages

• Among decedents admitted to high-intensity daytime staffed ICUs, the addition of a nighttime intensivist was associated with a reduced time between ICU admission and death.

• Among decedents admitted to high-intensity daytime staffed ICUs, the addition of a nighttime intensivist was associated with reduced odds of death at night.

• The addition of nighttime intensivist staffing to high-intensity daytime staffed ICUs may improve the quality of end-of-life care.

## Abbreviation

APACHE: Acute physiology and chronic health evaluation.

## Competing interests

The authors declare that they have no competing interests.

## Authors’ contributions

LAR contributed to the study design, performed analysis and interpretation of the data, and drafted the manuscript. DJW contributed to the analysis and interpretation of the data and revision of the manuscript. AEB contributed to the analysis and interpretation of the data and revision of the manuscript. JMK conceived the study and study design, assisted in the analysis and interpretation of the data, and revised the manuscript. All authors read and approved the final manuscript.

## Authors’ information

LAR is a pulmonary and critical care fellow at the University of Pittsburgh. DJW is an Assistant Professor of Critical Care Medicine and Emergency Medicine at the University of Pittsburgh. AEB is an Associate Professor of Medicine, Clinical and Translational Science, and Health Policy & Management at the University of Pittsburgh. JMK is an Associate Professor of Critical Care, Medicine, and Health Policy & Management at the University of Pittsburgh.

## Supplementary Material

Additional file 1: Table S1. presenting results of sensitivity analyses comparing ICUs with versus without nighttime intensivists. Click here for file
