# Supplementary material for: Nighttime intensivist staffing and the timing of death among ICU decedents: a retrospective cohort study
Source: Crit Care. 2013 Oct 3;17(5):R216. doi: 10.1186/cc13033 (PMC4057319; doi:10.1186/cc13033)
Supplement: Additional file 1: Table S1. — presenting results of sensitivity analyses comparing ICUs with versus without nighttime intensivists. [file cc13033-S1.pdf]

Additional Table 1. Sensitivity analyses results comparing ICUs with vs. without nighttime intensivists

Sensitivity Analysis	N	LOS in Days (95% CI)	p-value	OR for Death at Night (95% CI)	p-value
Original analysis	3553	-2.5 (-3.5, -1.5)	<0.001	0.75 (0.60, 0.94)	0.01
ICU LOS	3553	-1.4 (-2.2, -0.7)	<0.001	n/a	n/a
Including hospice patients	3711	-2.6 (-3.7, -1.6)	<0.001	n/a	n/a
Alternative definition of nighttime death (8pm-8am)	3553	n/a	n/a	0.76 (0.60, 0.96)	0.02

LOS = length of stay; CI = confidence interval; OR = odds ratio; ICU = intensive care unit; n/a = not applicable
